# Impact of Training of Primary Health Care Centers’ Vaccinators on Immunization Session Practices in Wasit Governorate, Iraq: Interventional Study

**DOI:** 10.2196/14451

**Published:** 2019-10-07

**Authors:** Ali Sadiq Amily, Faris Lami, Yousef Khader

**Affiliations:** 1 Immunization Section Wasit Directorate of Health Iraqi Ministry of Health Kut Iraq; 2 Department of Community and Family Medicine College of Medicine Baghdad University Baghdad Iraq; 3 Faculty of Medicine Jordan University of Science & Technology Irbid Jordan

**Keywords:** immunization, primary health care, intervention, practices, Iraq

## Abstract

**Background:**

Immunization averts more than 2.5 million deaths of children annually. The World Health Organization (WHO) and the United Nations Children’s Fund estimates of immunization coverage in Iraq in 2015 revealed a 58% coverage for the third dose of the diphtheria-tetanus-pertussis vaccine and a 57% coverage for the measles vaccine. High-quality immunization session practices (ISPs) can ensure safer, more effective vaccination and higher coverage rates.

**Objective:**

The goal of this study was to assess the impact of training of primary health care centers’ (PHCs) vaccinators on the quality of ISPs.

**Methods:**

This was an interventional study conducted on 10 (18%) PHCs in Wasit Governorate. Two PHCs were randomly selected from each health district. ISPs were assessed by direct on-job observation, using modified WHO immunization session checklists. Findings were grouped into seven domains: vaccine and diluent management, cold chain management, session equipment, registration, communication, vaccine preparation and administration, and waste management. The vaccinators were enrolled in a one-day training session using the WHO module, “Managing an Immunization Session”, and one month later a second assessment was conducted using the same tools and techniques. We then calculated the median differences of the domains' scores.

**Results:**

A total of 42 vaccinators were trained, with 25 (60%) of them having graduated from technical health institutes, but only 15 (36%) having had previous training on standard ISPs. Following training, a significant improvement was noticed in three domains: vaccines and diluents management (*P*=.01), cold chain management (*P*=.01) and vaccine preparation and administration (*P*=.02).

**Conclusions:**

The training of the PHCs' vaccinators for a single day was effective in improving some ISPs. We would recommend using this training module, or a more in-depth one, for other PHCs to improve utilization of immunization services.

## Introduction

Vaccination is one of the greatest public health achievements [1], with the greatest impact on human health and longevity compared to any other [2]. Apart from safe water, nothing other than vaccination, not even antibiotics, has had such a major effect on the reduction of mortality and on population growth [3]. Globally, vaccines prevent more than 2.5 million deaths of children each year [4], with one good example being the global reduction in deaths from measles by 79% between 2000 (651,600 deaths) and 2015 (134,200 deaths) [5].

Although vaccines are considered safe, they are not risk-free, and adverse events following vaccination may occasionally occur. Public trust in vaccine safety is key to the success of any vaccination program [1], thus, health care professionals must develop and maintain the highest possible competency with vaccination procedures. However, this process is becoming increasingly difficult due to a lack of resources and due to continuous pressure on professionals’ time. Knowledge is vital in maintaining comprehensive vaccination programs and strengthening best practices during daily work, and the goal is to use this knowledge to build vaccinators’ abilities and to improve immunization session practices (ISPs) [6].

Unfortunately, in Iraq, enrolling newly assigned vaccinators in routine training courses is not part of their training system, and while this might happen occasionally it is not scheduled. Thus, vaccinators can handle the challenges they may encounter during routine daily work by getting into close contact with senior vaccinators, at their sites, to gain further skills and build up their skills.

In addition to factors related to knowledge, skills, attitudes, and training of health care professionals, another factor that may play a role in strengthening vaccination competence is the vaccination environment. It should be quiet, safe, spacious, warm, private, and soundproof. However, excess amounts of work and staff shortages may weaken this competence [6].

World Health Organization (WHO) and United Nations Children’s Fund (UNICEF) estimates of immunization coverage in Iraq in 2015 revealed a 58% coverage for the third dose of diphtheria-tetanus-pertussis vaccine, a 63% coverage for the third dose of oral polio vaccine, and a 57% coverage for the first dose of measles containing vaccine [7] against a 95% national target for each antigen [8]. Ensuring high quality ISPs can lead to safer and more effective vaccination as well as higher coverage rates. Thus, it is of paramount importance to assess these practices and to ensure they are all the best quality, particularly when, according to our knowledge, such an assessment had never been done before in Iraq or in Wasit governorate.

The aim of this study was to assess ISPs in selected primary health care centers (PHCs) in Wasit governorate and to assess the impact of training of their vaccinators on the quality of ISPs.

## Methods

### Study Design and Setting

This interventional study was conducted in 10 PHCs in Wasit Governorate, one of the 18 governorates in Iraq. Wasit is in eastern Iraq, 160 km to the south east of the capital, Baghdad. It occupies an area of 17,153 km^2^, with a total population of 1,149,059. It has six health districts: Kut-1 (10 PHCs), Kut-2 (13 PHCs), Hai (10 PHCs), Suwayrah (13 PHCs), Numaniyah (3 PHCs), and Aziziyah (6 PHCs).

The 10 (18%) PHCs were selected by simple random sampling from the six health districts in Wasit Governorate. Two PHCs were randomly selected from each health district (except for Numaniyah and Aziziyah districts, as they were instead treated as a single district and only two PHCs were randomly selected from both). All the selected PHCs regularly provide immunization services.

We visited each of these PHCs once to assess ISPs with direct on the job observation, using modified WHO immunization session checklists [9]. Then, the vaccinators were enrolled in a one-day, UNICEF-funded training program using the WHO module *Managing an Immunization Session* [9]. The same tools and techniques were used again one month later to assess ISPs in the same PHCs.

### Immunization Session Checklists

We developed three checklists using Epi Info 7 (Centers for Disease Control and Prevention [CDC], Atlanta) based on the WHO immunization session checklist [9], the WHO checklists for vaccines and immunization [10], and the national guidelines for vaccines and immunization [8,11,12]. The developed checklists were a checklist for setting up the immunization session (see Multimedia Appendix 1), a checklist for the conduct of the immunization session (see Multimedia Appendix 2), and a checklist for concluding the immunization session (see Multimedia Appendix 3). These checklists were used to assess 62 ISPs.

### Immunization Session Practices

ISPs are the tasks a health worker needs to perform to ensure the quality of an immunization session. They covered the following aspects: preparation for the session, communication with clients and caregivers during each encounter, assessment of infants before vaccination, correct technique for giving vaccines, closing the session, and recording data [9]. Each of the 62 ISPs was given a score of one when correctly practiced, or zero if not. For analytic purposes, these ISPs were grouped into seven domains: Vaccine and diluent management (12 ISPs), cold chain management (6 ISPs), session equipment (7 ISPs), card review and registration (14 ISPs), communication with clients and caregivers (4 ISPs), vaccine preparation and administration (14 ISPs), and waste management (5 ISPs).

### Questionnaires

Two questionnaires were developed using Epi Info 7. The first was the vaccinator’s questionnaire, which was used to collect data on health care workers' (HCWs) age, gender, level of education (classified as intermediate school, high school, nursing school, technical health institute, college of nursing, and nonmedical college), number of years of working in the field of immunization, and if they received any formal training on standard ISPs. The second questionnaire was used to collect information from PHCs, such as the district’s name, the number of doctors in the PHC, the number of vaccinators working in the immunization unit, the number of people served by the PHC according to 2016 population, and the average daily number of people receiving vaccinations in the PHC.

### Training Program

Vaccinators in the selected PHCs were enrolled in a one-day training program that was conducted in the hall of the Center for Training and Development-Wasit Directorate of Health. A total of 42 vaccinators from the selected sites were trained after dividing them into two groups, with each group including half the number of vaccinators working in each PHC (in order to not create a shortage in the manpower working in the immunization unit in any of these PHCs on the day of training). The first group received training on Monday, November 28, 2016 and the second on Wednesday, November 30, 2016.

The training material was adopted from the WHO immunization training resource,*Managing an Immunization Session* [9]. We translated this module into Arabic and presented it as a PowerPoint show supported by three video clips about: (1) how to give the Bacille Calmette-Guerin vaccine to an infant; (2) the correct technique for giving intramuscular injections to infants; and (3) how to manage the immunization session as a whole. The presentation also had many pictures that were adopted from the CDC website.

Ethical approval was obtained from the Public Health Department at Wasit Directorate of Health, and UNICEF funded the training over its two days with an average cost of about 35 United States Dollars per vaccinator.

### Statistical Analysis

First, collected data were entered into Epi Info 7 (because the checklists and questionnaires were developed using the software), and then further processing and analyses were done using Excel (Microsoft, Redmond) to calculate the mean and median scores for each domain. Each single domain had a specific number of ISPs that were each given a score of one when correctly practiced or zero if not. After that, the mean and median for all practices contained in a single domain were calculated, giving a final score for that domain which ranged from 1-0 for the perfect domain and for the least achieving one, respectively. The above calculation was performed twice, once before and another time following the training. Then, the differences between pre- and posttraining domains' scores were calculated and the Wilcoxon signed-rank test was used to determine statistical significance. A *P*<.05 was considered statistically significant.

## Results

The study was conducted among 10 PHCs with 42 vaccinators. A total of 25/42 vaccinators (60%) had graduated from technical health institutes (two years following high school). All others had a lower education level, and none were college graduates. Only 15 (36%) of them had previous training on ISPs. The range of years of experience as vaccinators was 1-25 years, with a median of 3 years. Overall, 10 (24%) of the vaccinators were newly assigned, with less than one year of experience in the field of immunization. The main characteristics of the studied PHCs and vaccinators are shown in Tables 1 and 2, respectively.

Among the 10 studied PHCs, six (60%) showed a statistically significant improvement in immunization session practices following the training of vaccinators, two (20%) showed an improvement that was not significant, and the remaining two (20%) PHCs showed some unexpected decline in their ISP scores (Table 3).

**Table 1:** Characteristics of 10 primary health care centers in Wasit Governorate, 2016.

Primary health care center	District	Doctors, n	Vaccinators, n	Catchment area population, n	Vaccinators’ density (vaccinators/10,000 people)
Badr Ul-Kubra	Kut-1	3	4	33,934	1.2
Badra	Kut-1	4	4	15,605	2.6
Falahiyah	Kut-2	0	3	40,865	0.7
Al-Hakiem	Kut-2	2	3	11,500	2.6
Muwaffaqiyah	Hai	3	6	33,107	1.8
Asskary	Hai	3	5	22,307	2.2
Numaniyah	Numaniyah and Aziziyah	5	11	72,403	1.5
Ahrar	Numaniyah and Aziziyah	3	3	26,588	1.1
Mazraa	Suwayrah	3	2	10,660	1.9
Shuhaymiyah	Suwayrah	3	1	19,189	0.5

**Table 2:** Characteristics of vaccinators working in 10 primary health care centers in Wasit Governorate, 2016 (N=42).

Characteristics		Distribution, n (%)
**Sex**		
	Male	22 (52)
	Female	20 (48)
**Educational status**		
	Technical health institute	25 (60)
	Nursing school	14 (33)
	High school	1 (2)
	Intermediate school	2 (5)
**Service duration**		
	<1 year	10 (24)
	1-5 years	20 (48)
	>5 years	12 (28)
**Previous training on** **immunization session practices**		
	Trained	15 (36)
	Not trained	27 (64)

**Table 3:** The change in scores of immunization session practices following training of vaccinators in 10 primary health care centers in Wasit Governorate, 2016.

Primary health care center	Pretraining, mean (SD)	Posttraining, mean (SD)	Change, mean (SD)	*P* value
Badr Ul-Kubra	0.73 (0.45)	0.63 (0.49)	–0.10 (0.47)	.11
Badra	0.63 (0.49)	0.76 (0.43)	0.13 (0.50)	.045
Falahiyah	0.71 (0.46)	0.68 (0.47)	–0.03 (0.48)	.60
Al-Hakiem	0.60 (0.49)	0.84 (0.37)	0.24 (0.59)	.002
Muwaffaqiyah	0.68 (0.47)	0.84 (0.37)	0.16 (0.52)	.02
Asskary	0.76 (0.43)	0.84 (0.37)	0.08 (0.49)	.20
Numaniyah	0.56 (0.50)	0.66 (0.48)	0.10 (0.39)	.06
Ahrar	0.60 (0.49)	0.94 (0.25)	0.34 (0.48)	<.001
Mazraa	0.53 (0.50)	0.92 (0.27)	0.39 (0.49)	<.001
Shuhaymiyah	0.53 (0.50)	0.85 (0.36)	0.32 (0.47)	<.001
Total	0.632 (0.081)	0.795 (0.108)	0.163 (0.160)	.01

There were varying degrees of improvement among the domains following the training. Three domains that were originally among the lowest scoring showed the most significant improvement, including: vaccine and diluent management, cold chain management, and vaccine preparation and administration. Other domains with higher scores included: session equipment, waste management, and card review and registration. These later domains showed a less remarkable and statistically nonsignificant improvement. Despite originally being a mid-level domain with a mean 0.5 (SD 0.5), communication with clients and caregivers increased only a small, nonsignificant amount following the training (Table 4).

All ISPs, with the percentage of PHCs correctly practicing them as well as the difference following the training, are shown in Table 5.

**Table 4:** The change in scores of immunization session practices, by domains, following training of vaccinators in 10 primary health care centers in Wasit Governorate, 2016.

Domain	Pretraining		Posttraining		*P* value
	Mean (SD)	Median	Mean (SD)	Median	
Vaccine and diluent management	0.5 (0.1)	0.5	0.8 (0.1)	0.8	.01
Cold chain management	0.3 (0.2)	0.3	0.8 (0.3)	1.0	.01
Session equipment	0.9 (0.1)	0.9	0.9 (0.1)	0.9	.26
Communication with clients and caregivers	0.5 (0.5)	0.4	0.6 (0.4)	0.6	.67
Card review and registration	0.7 (0.1)	0.7	0.8 (0.1)	0.8	.13
Vaccine preparation and administration	0.6 (0.1)	0.6	0.8 (0.1)	0.8	.02
Waste management	0.8 (0.2)	0.8	0.8 (0.1)	0.8	.52

**Table 5:** Percentage of primary health care centers (PHCs) with standard immunization session practices (ISPs) in Wasit Governorate, 2016

Domains and ISP		PHCs with pretraining standard practices (%)	PHCs with posttraining standard practices (%)
**Vaccine and diluent management**			
	Vaccine quantity checked	100	100
	Vaccines out refrigerator in required quantity	50	60
	Vaccines out refrigerator in order	20	80
	Diluent quantity matched	50	60
	Diluent type matched	90	100
	Label checked	10	70
	Expiry date checked	10	70
	VVM^a^ checked	40	60
	Unopened vials returned to refrigerator	100	100
	Unopened vials returned to USE FIRST^b^ box	20	80
	Opened vials discarded	90	100
	Vaccine stock for next session	40	50
**Cold chain management**			
	Icepacks conditioned	0	80
	Freeze indicator checked	40	90
	Carrier conditioned icepacks	0	80
	Carrier vaccine vials in middle	70	70
	Carrier pad on top	30	70
	Carrier lid closed tightly	40	80
**Session equipment**			
	AD^c^ syringes collected	100	100
	Reconstitution syringes collected	90	90
	Safety box collected	100	100
	AEFI^d^ medications collected	40	50
	Permanent register collected	100	100
	Daily register collected	90	100
	New cards collected	100	100
**Card review and registration**			
	Review DOB^e^ and age	90	100
	Review vaccines previously received	70	100
	Review vaccines eligible for	90	90
	Permanent register ID^f^	100	100
	Permanent register DOB	100	100
	Permanent register address	0	10
	Permanent register date and dose of vaccine	100	100
	Permanent register date and dose of vitamin A	80	60
	Daily register vaccine dose	100	100
	Daily register vaccine information	20	40
	Card registered given vaccine	100	100
	Card marked date of next immunization	70	100
	Summary report	20	40
	Children missed vaccination listed	80	40
**Communication with clients and caregivers**			
	Client and caregiver greeted	70	60
	Messaged date of next visit	50	70
	Messaged AEFI	40	50
	Messaged what to do in AEFI	40	40
**Vaccine preparation and administration**			
	Wash hands with soap	10	60
	Vaccine prepared on clean table	90	90
	Reconstituted with correct type of diluent	100	100
	Reconstituted with correct quantity of diluent	90	100
	Reconstituted with new disposable needle and syringe	80	100
	Reconstitution needle and syringe disposed in safety box immediately	70	90
	Membrane or opening not touched	40	90
	Reconstituted vial in pad of vaccine carrier	20	60
	Fill syringe just before administration	100	100
	Alcohol not used	100	100
	Needle not touched	90	90
	Recommended technique	50	90
	Correct injection site	50	90
	Contraindication checked	0	10
**Waste management**			
	AD syringe disposed immediately	80	100
	AD syringe disposed without recapping	100	100
	Safety box within reach of Staff	80	100
	Safety box closed when full	100	100
	Safety box out of reach of children	30	20

^a^VVM: vaccine vial monitor.

^b^USE FIRST: a box in the refrigerator to which unopened vaccine vials with acceptable VVMs should be returned at the end of the session to be used first in the next session.

^c^AD: auto-disable.

^d^AEFI: adverse event following immunization.

^e^DOB: date of birth.

^f^ID: identification.

## Discussion

Six (60%) of the studied sites showed significant improvement in ISPs following the training of their vaccinators. The most significant improvement was in the domains that got the lowest scores during the first assessment, which included: vaccine and diluent management, cold chain management, and vaccine preparation and administration. The posttraining assessment revealed an overall small improvement in ISPs’ mean score, from 0.63 (SD 0.08) to 0.80 (SD 0.11), which might be related to what was concluded by one review evaluating the effects of educational meetings (eg, courses, conferences, lectures, workshops), in that any improvement they cause is most likely to be small [13].

All the studied PHCs were major ones (supposed to be run by physicians) and all were supplying immunization services through routine sessions on a regular basis. The observed number of vaccinators working in these sites is not relative to the number of people whom they serve. This might cause the quality of the provided health service to vary, because the imbalance in the supply, deployment, and composition of human resources may lead to a lack of equality in the provision of health services [14]. On the other hand, health care systems managing a balanced provider workload and staff mix may result in better patient care delivery [15].

Ten (24%) of the vaccinators were new to working in immunization units, with an average service duration of less than one year. Thus, it was expected that those vaccinators would be less competent as they had less practical work experience [6]. While training could improve their professional practice and the health care outcomes for their patients [13], lack of training for 27 (64.3%) of the vaccinators might seriously affect their competence, thus resulting in low quality practices.

The two PHCs that showed decline following training were further assessed to discover the causes behind it. One of these PHCs was unexpectedly supplied with a large quantity of influenza vaccine, and to dispense the vaccine as fast as they could the vaccinators decided to vaccinate everybody attending the PHC, regardless of their risk status for influenza. The posttraining assessment was implemented during this time, while many people were inside the immunization room with no control over their entry or presence, which negatively affected vaccinators’ performance. In the second PHC, two of the three trained vaccinators were immediately moved away following the training and were replaced by only one of the newly assigned staff. Thus, the immunization unit lost much of its workforce as well as the skills gained from the training, which had an obvious adverse effect on the vaccinators’ performance and on ISPs.

The three significantly improved domains were those dealing with aspects related to the vaccine itself: handling, storage, preparation, and administration. Practices under these domains might be considered (from vaccinators' perspectives) the most important and might have the most major consequences on the vaccinees. Other practices that might be considered of less serious impact showed a less remarkable, statistically nonsignificant improvement. The impact of educational meetings may be smaller for outcomes that health professionals may perceive as not having serious consequences for patients compared to outcomes that they may perceive as having moderately or highly serious consequences for patients [13]. On the other hand, our training was supported by three video clips as well as many pictures dealing with the practices belonging to the three most improved domains, which might help the vaccinators be more competent in these areas.

The WHO states that ice pack conditioning is a process that takes time and advanced planning, but cold chain surveys have shown that this practice is widely ignored [16]. This was the exact situation in our settings, revealing that ice pack conditioning was the most problematic among cold chain practices.

Adrenaline was not available in half of our sites even following the training, even though it is the responsibility of the health district to supply its PHCs with these adverse events following immunization (AEFI) medications. Thus, for this practice to improve it might require the health district to include these drugs in its priority list that could be bought from the local market when they are not centrally supplied by the department of pharmaceuticals and medical equipment, a measure that the system never allows a PHC to do independently. Thus, this practice might be considered a complex behavior (complexity of behavior may depend on whether there was a need for change by the individual, a communication change, or a change in systems and thus, whenever a change in the system is required to change certain behavior, the latter might be considered more complex) that is unlikely to change much [13].

In one study conducted in the United Kingdom, they found that communication strategies to date have lacked clear evidence of efficacy in vaccination settings [17]. In our settings, communication with clients and caregivers only improved a little with training, which is like the results of another study conducted in Vietnam where 83.3% of health care providers do not communicate about vaccination when giving an injection [18].

From all of the above, we can conclude that ISPs were doing variably in our immunization sites before training, but none were being practiced to standard. Training of vaccinators was effective in improving some practices, especially those dealing with the technical aspect of vaccination (ie, vaccine management, cold chain management, and vaccine preparation and administration). However, it is important to remember that the pre- and posttraining assessments were each conducted during a single visit in any of the selected sites, which might be a limitation of the study. In addition, the vaccine coverage rate was not included in the study even though it is an important variable that might be correlated to ISPs, leaving open the possibility for another study to be conducted.

Therefore, we would recommend this training, after some refinement, for other PHCs in Wasit governorate and on the national level, as well as for other countries or settings that use the Arabic language, to improve vaccinators’ abilities and thus ISPs.

## Appendix

Multimedia Appendix 1 Checklist for setting up the immunization session.

Multimedia Appendix 2 Checklist for the conduct of the immunization session.

Multimedia Appendix 3 Checklist for concluding the immunization session.

## Abbreviations

AD: auto-disable
AEFI: adverse event following immunization
CDC: Centers for Disease Control and Prevention
DOB: date of birth
EMPHNET: Eastern Mediterranean Public Health Network
HCW: health care worker
ID: identification
ISP: immunization session practice
PHC: primary health care center
UNICEF: United Nations Children’s Fund
VVM: vaccine vial monitor
WHO: World Health Organization
