# Supplementary figures and images for: Impact of Training of Primary Health Care Centers’ Vaccinators on Immunization Session Practices in Wasit Governorate, Iraq: Interventional Study
Source: JMIR Public Health Surveill. 2019 Oct 7;5(4):e14451. doi: 10.2196/14451 (PMC6803885; doi:10.2196/14451)

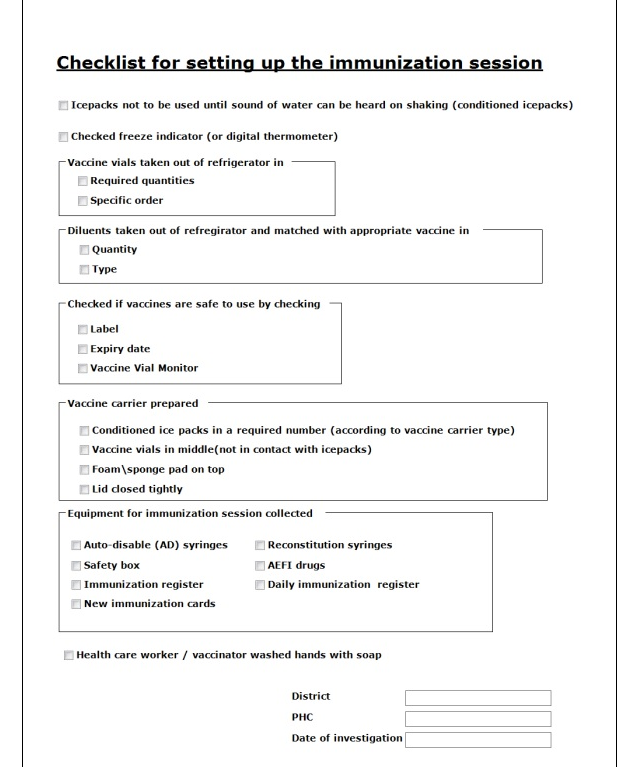

Supplement: Multimedia Appendix 1 [file publichealth_v5i4e14451_app1.png]

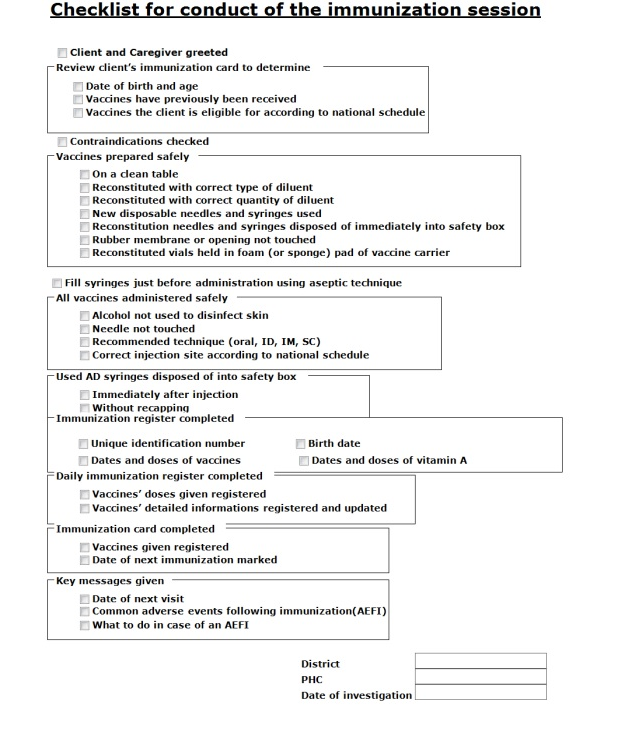

Supplement: Multimedia Appendix 2 [file publichealth_v5i4e14451_app2.png]

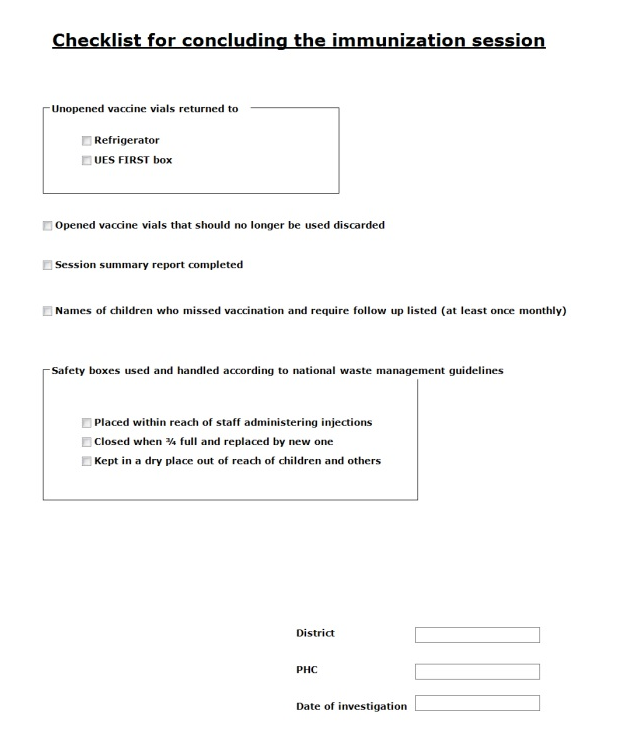

Supplement: Multimedia Appendix 3 [file publichealth_v5i4e14451_app3.png]
